# Sociological Theories to Explain Intimate Partner Violence: A Systematic Review and Narrative Synthesis

**DOI:** 10.1177/15248380231210939

**Published:** 2023-11-25

**Authors:** Sarah R. Meyer, Selina Hardt, Rebecca Brambilla, Shruti Shukla, Heidi Stöckl

**Affiliations:** 1 Institute of Medical Information Processing, Biometry and Epidemiology (IBE), Faculty of Medicine, LMU Munich, Munich, Germany; 2 Pettenkofer School of Public Health, Munich, Germany; 3 TUM School of Social Sciences and Technology, Technical University of Munich, Munich, Germany; 4 London School of Hygiene and Tropical Medicine, London, UK

**Keywords:** intimate partner violence, theory, sociology, neighborhood, family

## Abstract

Intimate partner violence (IPV) is a highly prevalent public health challenge and human rights violation. Sociological theories address social structures to understand prevalence and dynamics of IPV against women. This systematic review aims (1) to identify, describe, categorize, and synthesize sociological theories that account for predictors of IPV against women, and (2) to compare and contrast sociological theories of predictors of IPV against women. Following a structured search of nine electronic databases, members of the review team screened title/abstract and full texts against inclusion and exclusion criteria, to identify studies that engaged with theory/ies of predictors of IPV. Review team members extracted data according to a data extraction template developed for the review. Results are presented using a narrative synthesis approach. Following review of 108 included articles, included articles were grouped into sub-theories. The sub-theories provide differing, yet overlapping, accounts of predictors of male perpetration of IPV and women’s experience of IPV. Sociological theories primarily engage with exo- and macro-system levels of the social-ecological framework, yet some also address structural influences on individual behaviors. This systematic review fills a gap in theoretical syntheses of sociological theories of predictors of male-perpetrated IPV against women and also provides critical analysis of how these theories overlap and intersect. While sociological theories may not be able to fully explain all aspects of dynamics of male-perpetrated IPV against women, this overview indicates that there are several compelling components of sociological theory that hold explanatory power for comprehending how, where, and why IPV occurs.

## Introduction

Intimate partner violence (IPV) is the most common form of violence against women globally, with recent estimates indicating that nearly one in four women globally experience physical and/or sexual IPV in their lifetime (Sardinha et al., 2022). IPV is defined as acts perpetrated by a current or previous partner that cause physical, sexual, or psychological harm (WHO & PAHO, 2012). Over the past decades, the empirical evidence-base regarding the predictors of women’s experiences of IPV and male perpetration of IPV, especially in low- and middle-income countries (LMICs), has rapidly expanded. A range of theories have been employed to develop causal explanations as to why some women experience IPV and/ or why some men perpetrate IPV, why the prevalence of IPV varies across and between communities, countries, and regions, and how or why specific interventions work to prevent IPV (Beyer et al., 2015; Bourey et al., 2015; Capaldi et al., 2012).

Various ways of categorizing theories of predictors of IPV against women have been proposed. Gelles (1993) argued that three general classifications exist: individual models, which primarily employ psychological theories; sociological models, which often combine social and psychological explanations; and socio-structural models, which are primarily feminist theories. Ali and Naylor (2013) categorized explanations of IPV into biological, psychological, feminist, social, and ecological explanations of IPV. Ali and Naylor (2013) proposed that the “sociological perspective of IPV focuses on the social context and situations in which men and women live and where violence takes place,” shifting the focus on predictors of IPV from the individual perpetrator or the woman experiencing IPV to social structures, including the family (Dwyer et al., 1995). Synthesis of the range of sociological theories is particularly useful in LMIC contexts, where explanations for IPV centered around individual characteristics of perpetrators often contradict empirical evidence regarding the crucial role of social factors.

Reviews and syntheses of theories that address sociological theories exist, however, they all have limitations. Some reviews only focus on evidence published until 2010 (Lawson, 2012), omit key sociological sub-theories, such as neighborhood, peer relations, and criminological theories (Ali & Naylor, 2013; Lawson, 2012) or focus only on particular types such as intimate partner homicide (L. Graham et al., 2022). To provide a current synthesis of sociological theories addressing all forms of IPV against women, we conducted a systematic review and narrative synthesis of sociological theories of predictors of IPV against women. The objectives of this article are: (1) to identify, describe, categorize and synthesize sociological theories that account for predictors of IPV against women, and (2) to compare and contrast sociological theories of predictors of IPV against women.

## Methods

The following analysis is a sub-analysis of studies describing or employing sociological theories, based on a broader systematic review of all theories addressing predictors of male-perpetrated IPV against women (Prospero registration: CRD42022316584). We define predictors as variables, which may increase or decrease risk of women’s experience of IPV or men’s perpetration of IPV against women. We focus specifically on male perpetrators of IPV, and exclude studies that examined IPV perpetration without gender-disaggregation, or those focusing only on female perpetrators. Male and female-perpetration of IPV are different phenomena, which can be accounted for by different theoretical frameworks. Given the scope of the review, and the substantial breadth of sociological theories focused on male-perpetration, the present review focuses only on sociological theories accounting for male-perpetrated IPV.

### Search Strategy

The following electronic databases were searched, using a structured search strategy: PubMed, PsycINFO, Embase, CINAHL, Social Work Abstracts, Social Services Abstracts, ProQuest Criminal Justice, Web of Science, and Applied Social Sciences Index and Abstracts. The search strategy included two key aspects: (1) search terms for IPV, and (2) terms for theory, theories, and theoretical, specifying that the theory term had to be included in the title or abstract.

### Eligibility Criteria and Screening

Eligibility criteria are described in the registered protocol. Briefly, studies were included if they focused on theoretical discussions of predictors of IPV or on empirical research addressing one or more theories of IPV against women. Further inclusion and exclusion criteria are listed in Table 1. The title, abstract, and full text were screened using Rayyan systematic review software (Rayyan, 2022) and after removing duplicates. Two reviewers from the reviewer team screened each title, abstract, and full text. Where necessary, conflicts were resolved through discussions with the full team of co-authors. Two reviewers (S.R.M., S.H.) conducted the categorization of studies into particular theories and sub-theories, reading included full texts to determine the primary theory or theories addressed in the study, and labeling each included study with an over-arching theory (sociological theory, economic theory, feminist theory, psychological theory, biological theory, and dynamics and typologies of violence) and a sub-theory or sub-theories.

**Table 1.** Inclusion and Exclusion Criteria.

Inclusion Criteria	Exclusion Criteria
Focus is on theoretical discussion of predictors of intimate partner violence (IPV) OR focus is on empirical research addressing one or more theories of IPV against women (studies that include IPV alongside other forms of violence against women, such as non-partner violence, will be included)	Focus of study is consequences of IPV
Focus is on predictors of women’s experience of IPV and/ or predictors of men’s perpetration of IPV against women	Population of focus is same-sex couples
Theory must be addressed at some length (at least a paragraph) in the introduction or discussion	Outcome of interest is violence against men (studies including IPV against men will be included if IPV against women is also addressed)
Peer-reviewed publication	Outcome of interest is only violence against women that does not occur in intimate relationships
	Type of publication: gray literature, theses, systematic reviews

### Data Extraction and Analysis

For the purposes of the over-arching systematic review, we developed and piloted a data extraction template. Review team members tested the data extraction template by extracting data from the same four articles, and the template was then refined based on comparing data extraction results to ensure consistency. For studies with quantitative empirical data, data were extracted on research objectives, study design, sample size and sampling approach, country, key theoretical constructs, measurement approaches, primary findings, and interpretation in relation to theory. Based on the data extracted, we used narrative synthesis to explore and explain sociological theories of predictors of IPV. Narrative synthesis was chosen as it fits best with the review’s objectives and provides a way of bringing together findings from the included studies that utilizes words and text to “summarize and explain the findings of the synthesis” (Popay et al., 2006). While some recommendations exist to guide narrative synthesis for quantitative evidence (Ryan, 2013), there is no clear set of guidelines relevant to this systematic review, given its focus on theory rather than identifying intervention effectiveness, for example. As such, the narrative synthesis approach involved summarizing key elements extracted during data extraction, summarized each sub-theory, wrote the narrative based on these summaries and identified areas of intersection and overlap between theories.

## Results

**Figure 1.**
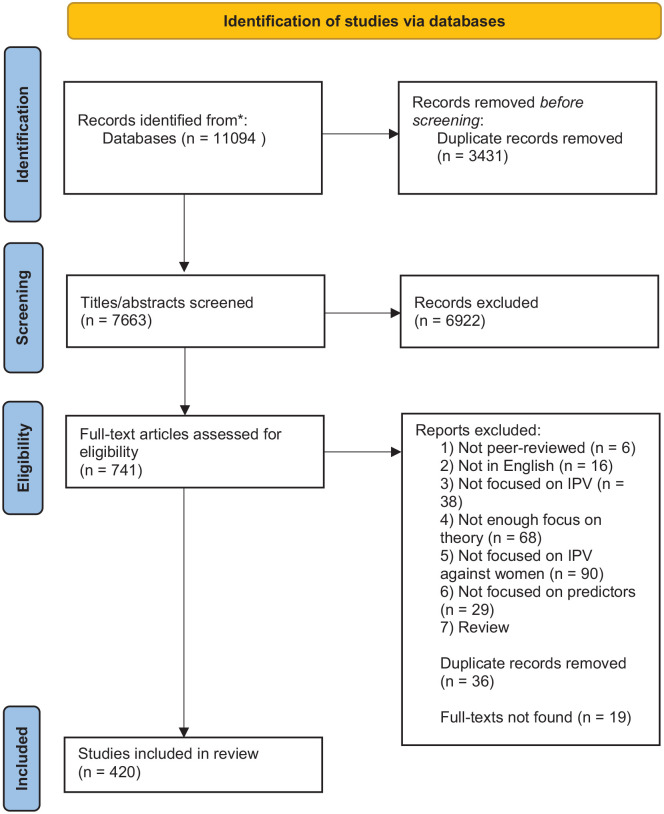
PRISMA [Preferred Reporting Items for Systematic Reviews and Meta-Analyses] 2020 flow diagram for new systematic reviews which included searches of databases and registers only.

Database searches for the overall systematic review including all theoretical approaches identified 7,663 unique records, of which 6,922 were excluded during title and abstract review (see Figure 1). The remaining 722 full texts were retrieved for full-text review, whereas 19 full texts could not be retrieved, following multiple efforts including searching on multiple databases and contacting authors directly. Of the 722 full texts, 420 articles addressing all theories of predictors of IPV were included after full-text review. Following theory categorization, 108 full texts were labeled as addressing sociological theories. Of the 108 full texts, 72 included quantitative empirical data.

Table 2 displays which sociological theory or theories each study addressed, as well as other non-sociological theories included in the study. Four included studies were published in the 1980s, 11 in the 1990s, 23 in the 2000s, 55 in the 2010s, and 16 thus far in the 2020s. Table 3 provides additional detail on studies which examined quantitative empirical data—research objectives, how these were related to theory and country in which the study was conducted. Of the 72 studies with quantitative empirical data, only 15 (21%) focused on or included LMIC countries.

**Table 2.** Included Articles and Theories.

Author	Year	Sociological Theory/Theories
Aborisade	2021	Criminological theory
Aghtaie	2018	Cultural theory
Ali	2008	Ecological theory
Anderson	2011	Criminological theory
Archer	2014	Peer theory
Baker	2018	Family theory
Beckmann	2021	Peer theory
Benson	2010	Criminological theory Cultural theory
Bogat	2005	Ecological theory Neighborhood theory
Bradley	2007	Cultural theory
Browning	2002	Criminological theory Neighborhood theory
Brownridge	2002	Ecological theory
Burge	2016	Family theory
Camargo	2019	Cultural theory
Carbone-Lopez	2012	Criminological theory
Chesworth	2018	Ecological theory
Chornesky	2000	Family theory
Cheung	2014	Criminological theory
Corradi	2016	Ecological theory
Cunradi	2010	Neighborhood theory Ecological theory
Cunradi	2014	Neighborhood theory
Daoud	2017	Neighborhood theory
Davis	2021	Ecological theory
DeKeseredy	1988	Peer theory
Douglas	2006	Criminological theory
Dwyer	1995	Ecological theory
Edwards	2014	Neighborhood theory
Ellis	1989	Cultural theory
Emery	2013	Criminological theory
Eriksson	2013	Criminological theory
Foshee	2011	Criminological theory
Frye	2012	Neighborhood theory
Fulu	2015	Ecological theory
Ghajarieh	2012	Ecological theory
Gibson	2001	Criminological theory
Gilfus	2010	Family theory
Goodrum	2004	Neighborhood theory
Goodson	2019	Neighborhood theory
Gracia	2015	Neighborhood theory
Graham	2017	Criminological theory Ecological theory
Graham	2020	Criminological theory
Grandin	1997	Cultural theory
Gul	2021	Cultural theory
Hackett	2011	Ecological theory
Hamptom	1994	Cultural theory
Hayes	2018	Criminological theory
Hayes	2022	Ecological theory Criminological theory
Heise	1998	Ecological theory
Hoffman	1994	Family theory
Hong	2010	Ecological theory
Huang	2001	Criminological theory
Iratzoqui	2019	Criminological theory
Jackson	2016	Neighborhood theory
Kerley	2008	Criminological theory
Kim	2012	Neighborhood theory
Krishnakumar	2021	Criminological theory
Lackey	1995	Criminological theory
Lackey	2003	Criminological theory
Lawson	1989	Family theory
Lawson	2012	Cultural theory Ecological theory
Lee	2010	Ecological theory
Lysova	2021	Cultural theory
Mannon	1997	Criminological theory
Marco	2018	Neighborhood theory
McCloskey	2016	Ecological theory
McQuestion	2003	Neighborhood theory
Morgan	2017	Neighborhood theory
Mulawa, Kajula	2018	Peer theory
Mulawa, Reyes	2018	Peer theory
Murray	2006	Family theory
Ngo	2022	Criminological theory
O’Neil	1997	Ecological theory
Outlaw	2015	Criminological theory
Parker	1990	Criminological theory
Pinchevska	2012	Neighborhood theory
Prandstetter	2022	Family theory
Pu	2021	Family theory
Ragetlie	2021	Ecological theory
Reckdenwald	2011	Criminological theory
Rodríguez-Menés and Safranoff	2012	Family theory
Roman	2012	Criminological theory Neighborhood theory
Rosen	2001	Family theory
Rotton	2001	Criminological theory
Saint-Eloy Cadely	2021	Family theory
Schubert	2002	Family theory
Schwartz	2001	Criminological theory Peer theory
Sedziafa	2016	Cultural theory
Showalter	2017	Neighborhood theory
Smith	1991	Peer theory
Smith	2016	Ecological theory
Spriggs	2009	Neighborhood theory
Stockdale	2012	Criminological theory
Straus	1994	Neighborhood theory
Taft	2009	Neighborhood theory
Tarzia	2021	Ecological theory
Taylor	2020	Neighborhood theory
Tolan	2006	Ecological theory
Van Wyk	2003	Neighborhood theory
Vezina	2011	Criminological theory Peer theory
Vezina	2015	Criminological theory
Voith	2017	Neighborhood theory
Waltermauer	2007	Criminological theory
Wick	2017	Criminological theory
Williams	1989	Criminological theory
Wright	2010	Cultural theory Criminological theory
Xie	2012	Criminological theory
Zavala	2022	Criminological theory
Zosky	1999	Family theory

**Table 3.** Empirical Studies: Research Objective(s) and Setting.

Author (year)	Research Objective(s)	Setting
Anderson (2011)	To investigate how job-related stress affects the likelihood that police officers use physical violence against an intimate partner	Baltimore, United States
Archer (2014)	To assess the association between physical aggression and self-control and cost-benefit assessment	Two studies: Aizwal, India and Salamanca, Spain
Baker (2018)	To explore the association between self-control and emotional and verbal aggression during couple conflict resolution	United States, Mid-Western university
Beckmann (2021)	To investigate how classroom normative climate regarding the perpetration of teen dating violence was related to adolescents’ self-reported perpetration of dating violence in the past 12 months	Lower Saxony, Germany
Benson (2010)	To address the question of why African Americans appear to have a higher likelihood of engaging in domestic violence than whites; to examine whether any significant individual-level relationship between race and intimate assault is rendered non-significant once controls are introduced for ecological context	United States, national study
Bogat (2005)	To examine the effects of community violence on women’s experiences of domestic violence and their mental health outcomes	Midwestern city, United States
Bradley (2007)	To examine whether or not former service in the military contributes to intimate relationship violence, controlling for combat exposure, relationship stressors and other statistical controls	United States, national study
Browning (2002)	To test whether a community’s structural characteristics, including concentrated disadvantage, residential stability, and ethnic heterogeneity, are associated with the level of partner violence, above and beyond individual, relationship, and network level factors; if there are any key neighborhood level factors associated with partner violence and whether these mechanisms mediate the association between structural characteristics and partner violence; and whether communities influence the likelihood of women reporting partner violence	Chicago, United States
Brownridge (2002)	To investigate the prevalence and causes of violence against immigrant women in Canada	Canada, national study
Burge (2016)	To assess whether models of partner aggression (cycle of violence, family systems theory, Duluth model) fit with three patterns of system dynamics (periodic, chaotic, random)	United States
Camargo (2019)	To examine the correlation between intimate partner violence and the type of domestic decision-making	Bolivia, national study
Carbone-Lopez (2012)	To describe incarcerated women’s involvement in intimate relationships with partners who are also engaged in criminal behavior; and to assess the effects of women offenders’ intimate relationships with criminal partners on their risk of IPV, net of other risk factors, including their own criminal behavior	Minnesota, United States
Cheung (2014)	To explore whether the degree of self-control of the husband is associated with his perpetration of violence against his wife	Hong Kong
Daoud (2017)	To examine the impact of neighborhood environment on the ethnic gap in IPV between Arab and Jewish women in Israel	Israel, national study
Davis (2021)	To examine whether the overall well-being of a country during one’s childhood affects the perpetration of domestic in emerging adulthood, and whether national status of women during childhood moderates the association between overall well-being of a country during childhood and domestic violence perpetration in emerging adulthood	19 countries
Douglas (2006)	To investigate the extent to which the criminogenic theory of corporal punishment (CP) operates at the societal level in ways that are parallel to the link between CP and violence found at the individual level	19 countries
Edwards (2014)	To examine how much community-level poverty rates and collective efficacy influence individual reports of IPV perpetration, victimization, and bystander intervention.	16 rural counties across eastern United States
Emery (2013)	To examine whether men’s current abuse of a child moderates the relationship between their patriarchal beliefs and current perpetration of marital violence	South Korea, national study
Foshee (2011)	To examine whether family, peer, school, and neighborhood risk and protective factors impact violence profiles	Rural counties in North Carolina, United States
Ghajarieh (2012)	To evaluate the factors that influence domestic violence against women in Neyriz and Estahban cities, Iran	Neyriz and Estahban, Iran
Gibson (2001)	To explore the generality of general strain theory, examining IPV perpetration in a sample of male police offices	Baltimore, United States
Goodrum (2004)	To examine the relationship between substance use and violence across rural-urban and Appalachian residences in a sample of incarcerated males with a history of substance abuse	Kentucky, United States
Goodson (2019)	To explore the associations between social disorganization, women’s absolute status, gender inequality, family violence, and forcible rape	Texas, United States
Gracia (2015)	To analyze the influence of neighborhood-level characteristics on small-area variations in IPV risk using spatial data on IPV cases and a Bayesian random-effects modeling approach	Valencia, Spain
Grandin (1997)	To compare national incidence rates of physical IPV between United States and Canada	United States and Canada, national studies
Hackett (2011)	To explore the relationship between a state’s level of development and the rates of IPV and intimate partner homicide in India	India, national survey
Hamptom (1994)	To examine prevalence and risk factors for IPV in a representative sample of African-American families	United States, national survey
Hayes (2018)	To test if the presence of the victim’s friends/family, the abuser’s friends/family, or a bystander during a physical abuse incident or threat of physical abuse affects the likelihood of repeat victimization	Chicago, United States
Hayes (2022)	To develop individual- and country-level indicators of opportunity to understand the experience of IPV among married women in the Global South	41 countries, Demographic and Health Surveys—national surveys
Hoffman (1994)	To assess use of physical violence by Thai men against women drawing from resource, structural, and social psychological theories	Bangkok, Thailand
Huang (2001)	To examine the causes and consequences of IPV in the African American population, as well as comparing patterns men and women	North Carolina, United States
Iratzoqui (2019)	To examine the associations between child maltreatment, dating violence, and intimate partner victimization	United States, national survey
Jackson (2016)	To examine how residential neighborhood influences women’s IPV risk	Chicago, United States
Kerley (2008)	To assess the effects of low self-control on offending and victimization among women in Bangkok, Thailand	Bangkok, Thailand
Kim (2012)	To examine the extent to which selected neighborhood characteristics were associated with IPV against women	Santiago, Chile
Lackey (1995)	To explore if successful bonding experiences in adulthood differentiate persons with a violent family history who are nonviolent from those who perpetuate IPV against their partners	United States, national survey
Lackey (2003)	To examine how informal social control can explain continuity and change in the impact of family of origin violence on later IPV	United States, national survey
Lysova (2021)	To test cultural spillover theory as it applies to IPV in a multinational context	33 countries
Marco (2018)	To analyze the influence of university campuses on IPV risk	Valencia, Spain
McQuestion (2003)	To examine household and neighborhood effects jointly on physical and sexual IPV in Colombia	Colombia, national study
Morgan (2017)	To examine Chicago neighborhoods and Illinois counties to determine the effects of social disorganization measures on IPV	Illinois, United States
Mulawa, Reyes (2018)	To assess the degree to which peer network gender norms are associated with Tanzanian men’s perpetration of IPV and whether the social cohesion of peer networks moderates this relationship	Dar es Salaam, Tanzania
Ngo (2022)	To examine gender differences in IPV perpetration, exploring if gender differences in crime (IPV perpetration) are due to differences between males and females in their standing on the life domains or differences in the effect of the life domains on IPV perpetration among males and females	United States, national survey
Outlaw (2015)	To examine the applicability of the routine activity factor, in particular, guardianship, to IPV	United States, national survey
Parker (1990)	To find different variables associated with IPV, using both the macro-social approach to homicide and micro-social approach to IPV	299 metropolitan cities, United States
Prandstetter (2022)	To test the role of couple dissatisfaction as a mediator between IPV victimization and parental burnout, and the link between IPV victimization and dysfunctional parenting	Austria
Pu (2021)	To understand the co-occurrence of parent–child aggression risk, IPV victimization and child behavior problems by conducting longitudinal analysis	Southeastern city, United States
Ragetlie (2022)	To investigate the association between household food production and IPV in Atacora, Benin	Atacora, Benin
Reckdenwald (2011)	To explore how various predictors are associated with the changing nature of gender-specific intimate partner homicide	178 urban cities, United States
Rodríguez-Menés and Safranoff (2012)	To test five theoretical explanations, including family systems theory, to assess the association between sociodemographic variables and IPV	Spain
Roman (2012)	To test whether the density of alcohol outlets across neighborhoods is positively associated with police calls for IPV	Washington D.C., United States
Rosen (2001)	To examine dating relationships from the perspective of social learning theory and Bowen family systems theory	United States
Rotton (2001)	To examine association between time of day, day of week, season and temperature and IPV	Minneapolis, United States
Saint-Eloy Cadely (2021)	To examine whether interparental aggression in adolescence, more social-information processing biases, higher levels of relationship insecurities, and less discontinuity (greater stability) in romantic relationship predict membership active aggression as compared to minimal aggression	Four cities, United States
Schubert (2002)	To examine characteristics of men who perpetrate IPV through Bowen’s theory of differentiation, and how much egalitarian attitudes relate to IPV	Men’s counseling groups, United States
Schwartz (2001)	To test feminist routine activity theory with a large representative sample of undergraduates on a Canadian college campus to improve the theory’s explanatory value	Canada, national survey
Sedziafa (2016)	To examine what influences physical, sexual, and emotional violence among matrilineal and patrilineal kin groups in Ghana	Ghana, national survey
Showalter (2017)	To test the hypothesis that social cohesion and informal social control are associated with lower rates of IPV	20 large cities, United States
Smith (1991)	The study is an exploratory examination of the peer support thesis	Toronto, Canada
Spriggs (2009)	To explore how family and school disadvantage relate to dating violence victimization	United States, national survey
Straus (1994)	To determine societal characteristics (social stratification/inequality and social integration vs. disorganization) that predict male IPV perpetration	United States, national survey
Taylor (2020)	To examine the association between local levels of violent crime and adolescent relationship aggression (perpetration and victimization)	United States, national survey
Van Wyk (2003)	To investigate the direct and interactive effects of social disorganization variables on IPV against women	United States, national survey
Vezina (2011)	To examine whether a risky lifestyle mediates the relationship between deviant peer affiliation and adolescent girls’ experience of dating violence	Quebec, Canada
Vezina (2015)	To examine the prevalence of dating victimization patterns and the associations between such patterns and family, peer, and individual variables	Quebec, Canada
Voith (2017)	To examine (1) if levels of male perpetration of IPV vary across census tracts, and (2) if so, whether neighborhood-level factors (social disorganization and collective efficacy) are associated with IPV prevalence	16 cities, United States
Waltermauer (2007)	To examine the impact of residential change on a woman’s risk of IPV and non-partner sexual violence	United States, national survey
Wick (2017)	To explore how online behaviors may make college students vulnerable to cyber IPV	Large Southwestern state, United States
Williams (1989)	To examine the role of attachments, commitments, involvement, and beliefs in male-perpetrated IPV.	United States, national survey
Wright (2010)	To test whether the immigrant paradox (i.e., lower crime rates in areas with higher levels of immigrant concentration) can be extended to IPV	Chicago, United States
Xie (2012)	To examine the impact of women’s status on IPV victimization and non-partner violence	40 metropolitan cities, United States
Zavala (2022)	To explore whether religious involvement can reduce IPV victimization	United States, national survey

### Narrative Description of Sub-theories

Following review of all included articles, included studies were categorized into the following sub-theories: criminological theory (38 articles), neighborhood theory (27 articles), cultural theory (11 articles), family theory (12 articles), and peer theory (7 articles). Finally, we describe the ecological framework (24 articles), which is a way to integrate these theories into a multilevel framework of predictors of IPV. Table 4 summarizes key tenets of each sub-theory, as well as highlighting overlap and intersections between sub-theories.

**Table 4.** Intersections Between Sociological Theories.

Theory/Sub-Theory	Key Tenet	Ecological Level(s)	Intersection with Other Sub-Theories
Criminological			
Routine activity theory	• Daily activities create opportunities for crime • Crime of violence against women requires motivated offender, suitable victim and lack of capable guardian	Individual level Micro-system	• Social disorganization—neighborhood structures can influence daily activities, that is, availability of alcohol outlets; capable guardianship can be impacted by spatial factors at neighborhood level • Peer support—peer attitudes and actions influence actions of motivated offender
Strain theory	Stress can trigger negative emotions, which result in deviant behaviors • Strain can emerge from failure to achieve positively valued goals, loss of positively valued stimuli (i.e., employment or social support) and experience of negatively valued stimuli (i.e., experience of abuse)	Individual level Micro-system Exosystem	• Peer theory—peer beliefs and behavior can shape individual perceptions of appropriate emotional response to strain
Self-control theory	• Limitations in capacity for self-control are associated with criminal behavior, including IPV perpetration • Focus on the perpetrator, whose low self-control is characterized by impulsivity, poor planning, and lack of insight into consequences	Individual (primarily) micro-system (family can shape levels of self-control)	
Social bond theory	• Deviant behavior is the norm • Social bonds can lead people to refrain from crime	Individual Micro-system Exosystem	• Peer theory—strong bonds to peers can protect against violence perpetration • Neighborhood theory—social controls against IPV perpetration can be enforced by social bonds at the neighborhood level
Neighborhood theory			
Social disorganization theory	• Factors such as socioeconomic disadvantage may decrease a sense of community, limiting exertion of social control	Exosystem Macro-system	• Routine activity theory—social disorganization can erode community capacity to provide effective guardianship
Collective efficacy theory	Collective efficacy is social cohesion that can mitigate impacts of social disorganization	Exosystem Macro-system	• Social bonds—can be a form of collective efficacy • Routine activity theory—collective efficacy can support capable guardianship
Social contagion theoryc	Community violence and IPV may be linked by “contagion” of one form of violence in one sphere to another	Exosystem Macro-system	• Cultural spillover—social contagion is one form of cultural spillover
Cultural theory			
Sub-culture of violence theory	• Social and gender norms associated with IPV (i.e., patriarchal norms) can form a sub-culture of violence Levels of other forms of violence within spatial area or other grouping (i.e., class or race) can impact level of IPV	Macro-system	• Social disorganization—elements of neighborhoods, that is, high immigrant concentration, may prevent or result in higher levels of IPV, depending on the sub-culture of violence within these groups • Culture of violence can operate a neighborhood level, as well as within class, race or religious groupings
Cultural spillover theory	• Where violence is endorsed/accepted in a specific sub-culture/context, this legitimization of violence can be generalized to other aspects of life, that is, intimate relationships	Macro-system	• Social contagion—social contagion is one form of cultural spillover
Family theory	• Family is a system which nested sub-systems that can influence IPV perpetration • Linkages between child victimization and IPV perpetration within families	Micro-system	• Cultural spillover—norms and experiences at the family-level can spillover between parents to result in violence against children, and subsequent perpetration of IPV Self-control theory—lower self-control in partners can lead to aggression and violence
Peer theory	• Where peers endorse or support IPV perpetration, individual men will be more likely to perpetrate IPV	Exosystem	• Sub-culture of violence/Cultural spillover—peer group could be one group that exhibits a particular culture of violence, which impacts individual behavior

#### Criminological Theory

Criminological theories focus on the behavior of perpetrators of specific crimes to understand motivation and circumstances that allow for these behaviors (Okada, 2011). Criminological theories were addressed in 38 articles. Initially, IPV was rarely explained using criminological theories as IPV researchers had thought that “domestic violence is different from crime” (Williams & Hawkins, 1989). Over the past three decades, researchers have drawn on and adapted criminological theories to understanding IPV.

##### Routine Activity Theory

Routine activity theory considers the role of daily activities in creating opportunities for potential perpetrators and victims to interact, possibly increasing the risk of male perpetration and female victimization. Routine activity theory focuses on three components, which enable crime: a potential offender, a suitable target (i.e., activities that might make women more susceptible to IPV, for example, drug or alcohol use), and the absence of a capable guardian (i.e., a person who is willing and able to intervene or can deter the criminal from acting) (Bottoms & Wiles, 1997; Hayes, 2018; Outlaw, 2015). This shifts the emphasis from a traditional criminological focus on “firm motives and rational planning” to recognition of acts of violence, such as IPV, that may be characterized by “lack of clear motives and careful planning” (Mannon, 1997).

Several studies applied routine activity theory specifically to male perpetration of IPV in various contexts and participants, such as LMICs, adolescents, metropolitan cities, and college students (Carbone-Lopez & Kruttschnitt, 2010; Egbert & Muniz, 2022; Hayes, 2022; Krishnakumar & Verma, 2021; Parker & Toth, 1990; Vezina et al., 2011; Vézina et al., 2015; Xie et al., 2012). For instance, Carbone-Lopez and Kruttschnitt (2010) found support for routine activity theory among incarcerated women; in that, involvement in risky activities and being in a relationship with someone also involved in crime was associated with higher levels of IPV experience. However, longitudinal analysis of changes in women’s status and violence risk in U.S. metropolitan cities showed that routine activity theory was supported for non-partner violence, but not for IPV (Xie et al., 2012).

Several studies focused on how guardianship within routine activity theory may be relevant specifically in the case of IPV. Schwartz et al. (2001) argued that routine activity theory needs to better account for the role of motivation of potential offenders. Their analysis of representative data from college campuses in Canada found that men who reported that male peers supported dating violence were more likely to have perpetrated sexual dating violence in the past 12 months (Schwartz et al., 2001). One study found that in contexts of control-motivated IPV, presence of more adults or children in the household, potential guardians of women who may be at risk of violence, increases IPV risk rather than reducing it, because they serve as an audience for displays of power and control (Outlaw, 2015).

Other studies employed theoretical constructs related to routine activities theory, such as Waltermaurer’s examination of residential change, which, she argues, affects “structurally and individually, the risk both of offending and of victimization” (Waltermaurer, 2007). Graham et al. (2017) employed situational crime theory, which extends routine activity theory to include environment, for example availability of alcohol outlets and crime precipitators, for example cues from others that violence is acceptable. One study combined routine activities theory and social disorganization theory, finding that off-premises alcohol outlets had a strong significant relationship to domestic violence-related police calls in the District of Columbia, United States (Roman & Reid, 2012). Another study showed that routine activity theory can be used to explain the curvilinear association between temperature and IPV through time of day, for example, less IPV occurred at the hottest time of the day compared to evenings (Rotton & Cohn, 2001).

##### Strain Theory

Strain theory is another commonly discussed sub-theory, which posits that stress can trigger negative emotions, for example, anger and maladaptive coping through deviant behavior, such as perpetrating IPV (Agnew, 1992; Iratzoqui & Watts, 2019). Two studies found that police officers’ exposure to stressful events in the workplace was associated with increased use of physical IPV (Anderson & Lo, 2011; Gibson et al., 2001). Anderson and Lo (2011) found that authoritarian spillover and negative emotions mediate this relationship. Iratzoqui and Watts (2019) operationalized strain as child maltreatment. They found that it was associated with increased risk of experiencing dating violence or IPV among youth in the United States, and that this association was mediated by negative emotions (Iratzoqui & Watts, 2019).

##### Self-Control Theory

Self-control theory emerged from the work of Gottfredson and Hirschi (1990) and suggests that self-control is shaped by family socialization and that limitations in capacity for self-control are associated with criminal behavior, including IPV perpetration. Specifically, low self-control is characterized by impulsivity, poor planning, and lack of insight into consequences. Among men residing in Johannesburg, South Africa, IPV perpetration was associated with executive control dysfunction and behavior dysregulation (Bantjes et al., 2020). One study combined strain and self-control theories, finding that strain (i.e., childhood physical abuse and an unsatisfactory marital relationship) was significantly associated with male perpetration of physical and psychological IPV and that higher self-control was protective against IPV perpetration among men in Hong Kong (Cheung et al., 2014).

##### Social Bond Theory

Social bond theory was proposed by U.S. sociologist Hirschi in 1969 and proposes that strong social bonds prevent people from engaging in deviant behavior, such as IPV perpetration (Hirschi, 1969). Social bonds include attachment to others, involvement in work and social activities, moral beliefs and commitment to social goals, and personal aspirations (Williams & Hawkins, 1989). Lackey and Williams (1995) found that two aspects of social bonds—attachments and beliefs—were significantly associated with the probability of non-violence toward partners. In a longitudinal study on the intergenerational transmission of violence, Lackey utilized the age-graded theory of social control, a variation of social bond theory (Sampson & Laub, 1992). They found that among men, victimization by parents in adolescence was linked to lower levels of commitment to both partner and work, which significantly predicted later IPV perpetration (Lackey, 2003). Conversely, Williams and Hawkins (1989), using longitudinal data from the United States, found that attachment to significant others and beliefs is predictive of male non-aggression (Williams & Hawkins, 1989).

#### Neighborhood Theory

Neighborhood theories, when applied to predictors of male IPV perpetration as discussed in 27 included articles, consider the neighborhood a key macro-level social structure and argue that neighborhood-level factors influence individual-level risk (Jackson, 2016; Taft et al., 2009). Neighborhood-level factors may be structural, for example, level of crime or poverty at the neighborhood level; physical, for example, accessible green space or cleanliness of the neighborhood; or social, for example, social cohesion and trust among residents of the neighborhood (Kim et al., 2013). These factors can deter or promote violence, for instance, depending on neighborhood resources and processes, norms regarding the role of women, and women’s access to resources (Jackson, 2016; Sampson et al., 1997; Taylor et al., 2020). Neighborhood theories were developed to explain variation in levels of violence and crime across neighborhoods, primarily in the United States and have been less commonly applied in studies conducted outside of high-income countries (Pinchevsky & Wright, 2012). Neighborhood theories include *social disorganization, collective efficacy*, and *social contagion theories*.

Over the past two decades researchers have extended *social disorganization theory*, which has addressed public crimes and forms of violence such as gun violence and gang activity for nearly a century, to “private” forms of violence, including IPV, primarily in urban settings of high-income countries (Benson et al., 2003; Browning, 2002). Browning (2002) has argued that applying social organization theory to understanding male-perpetrated IPV is warranted given the role of collective efficacy in monitoring and sanctioning IPV. Social disorganization theory proposes that structural and material factors may decrease a sense of community and “collective ability,” such that a community struggles to “realize common values and address community problems” (Taylor et al., 2020), regulate residents’ behavior (Spriggs et al., 2009), and control or reduce crime (Goodson & Bouffard, 2019). Therefore, factors such as low socioeconomic status, residential instability, family disruption, and ethnic heterogeneity can indirectly affect crime rates (Goodson & Bouffard, 2019).

In contexts with high prevalence of adverse social factors, institutions that typically promote social control, including family, churches and schools, may lose their ability to exert social control over criminal behavior and violence, as well as diminish collective efficacy (Benson et al., 2004). Several national and regional studies in the United States found support for social disorganization theory, with measures of social disorganization at the state level associated with higher levels of IPV (Benson et al., 2004; Straus, 1994). Edwards et al. (2014) found that across 16 rural counties in the Eastern United States, community-level poverty predicted male-reported perpetration and female-reported IPV experience. Social disorganization theory proposes that alcohol use, a strong predictor of IPV against women, is one form of “deviant” behavior that may be more prevalent in neighborhoods with concentrated disadvantage, and that presence of alcohol outlets may interact with other neighborhood factors to increase IPV risk. A few studies conducted in high-income countries found partial support for some dimensions of social disorganization theory, for example residential immobility social disorganization variables were found to have significant effects on risk of IPV prevalence (Goodson & Bouffard, 2019; Gracia et al., 2015; Voith & Brondino, 2017) or only on intimate partner homicide (Browning, 2002). Kim et al. (2013) hypothesized that neighborhood disadvantage and IPV in Santiago, Chile may be associated through elevated social stress, finding that some characteristics of neighborhood social disorganization, for example, neighborhood trash levels, were associated with increased IPV prevalence.

In Sampson et al.’s (1997) seminal work, *collective efficacy* is defined as “social cohesion among neighbors combined with their willingness to intervene on behalf of the common good.” They propose that collective efficacy can mediate or mitigate neighborhood-level disadvantage. Variation in levels of collective efficacy partially explains variation in levels of violence across neighborhoods (Sampson et al., 1997). Concentrated disadvantage and high levels of residential instability can influence effective social control and therefore collective efficacy. Daoud et al. (2017) noted that collective efficacy may function differently for different ethnic groups, suggesting that this could be explained by neighborhood social capital—ties of trust, reciprocity, and norms between neighbors and community members. However, in their study of ethnic differences in IPV prevalence between Arab and Jewish populations in Israel, they found that collective efficacy was not associated with IPV prevalence and did not explain the ethnic gap in IPV prevalence between groups (Daoud et al., 2017). Edwards et al. (2014) and Daoud et al. (2017) found support for social disorganization theory, but not collective efficacy, as predictors of IPV. It has been suggested that this may be due to IPV being a fundamentally different form of violence and crime than other forms explained by collective efficacy theory (Frye & Wilt, 2001).

Edwards et al. (2014) found that collective efficacy was positively related to frequency of IPV bystander intervention, thus documenting an important mechanism through which collective efficacy may result in lower levels of male-perpetrated IPV. One study found support for a social interaction effect in analysis of Demographic and Health Survey data in Colombia; residing in an area with higher than average level of IPV significantly impacted experience of physical and sexual IPV, above individual-level risk factors (McQuestion, 2003). Daoud et al. (2017) found that linking social capital—women’s active participation in groups—and social support were protective against IPV, identifying potential constructs that may act to buffer neighborhood adversity and its impact on IPV.

Bogat et al. (2005) proposed another neighborhood theory, *social contagion theory*, which provides an explanation for the linkages between community violence and IPV, in that violence “may act as a contagion,” through social norms that accept and promote use of various forms of violence or through creation of an environment where violence is seen as an acceptable means of solving conflicts. Where social contagion theory is applied to explain male-perpetrated IPV, studies have specifically examined how or if other forms of violence are associated with IPV against women. However, Bogat et al.’s (2005) analysis of the association between community violence and IPV and Taylor et al.’s (2020) examination of the association between neighborhood crime rates and self-reported IPV experience and/ or perpetration both did not find evidence for social contagion theory.

#### Cultural Theory

Eleven included studies examined predictors of male-perpetrated IPV through the lens of culture. Sociological theories of the role of culture in explaining IPV can be divided into (1) studies that examine cultural norms, including patriarchy, and explore how these norms may influence male perpetration of IPV or increase women’s risk of IPV experience, and (2) studies that employ “cultural spillover” theory to explain IPV.

Among studies that examined the role of cultural norms, Aghtaie et al. (2018) employed the concept of “cultural violence” to explain how violence can become normalized in society, and how practices and values embedded within social constructs such as masculinity can encourage male perpetration of IPV. Camargo analyzed IPV in Bolivia using a DHS dataset, with results supporting the hypothesis that IPV is correlated with “patriarchal-type family structure” (Camargo, 2019). Sedziafa and Tenkorang (2016) utilized a DHS dataset in Ghana to explore how cultural factors related to specific kinship affiliations were associated with female IPV experience, finding that physical and sexual IPV are higher in patrilineal compared to matrilineal groups.

##### Culture of Violence, or Subculture of Violence, Theory

Culture of violence, or subculture of violence, theory extends the perspective on the role of norms in impacting levels of male IPV perpetration. Culture of violence theory, proposed by Gelles and Straus (1979) in relation to IPV, argues that variations in the distribution of violence can be explained by background cultural characteristics, for example, in contexts where other forms of violence are highly prevalent, IPV perpetration will be more prevalent. Ellis discussed patriarchal norms associated with IPV as a “sub-culture,” and identified interactions with peers who are “the carriers, transmitters, and supporters of patriarchal subcultural values and norms legitimating male dominance” as particular risk factors for male perpetration of IPV (Ellis, 1989). Wolfgang and Ferracutti (1967), who developed subculture of violence theory to explain patterns of interpersonal violence more broadly, argued that values prevalent in specific subcultures operate to make violence more likely, as acceptance of violence is passed on and promoted by group members. Similarly, Grandin and Lupri (1997) compared IPV data from Canada and the United States, testing the hypothesis that given a higher background of community violence in the United States, levels of IPV would be higher in the United States than in Canada if culture of violence theory was supported, a hypothesis that was disproven. Gul et al. (2021) explored culture of honor, a set of beliefs about the importance of personal reputation, and its role in driving differing levels of IPV across regions of the United States, noting that a predominant culture of honor could drive justification of male-perpetrated IPV in cases where a male intimate partner perceives the female’s behavior as bringing shame, or romantic rejection as being a legitimate reason for violence (Gul et al., 2021).

##### Cultural Spillover Theory

Cultural spillover theory, first proposed by Baron and Straus (2014), suggests that where a specific culture or sub-culture valorizes and endorses violence perpetration, this legitimization of violence is likely to be generalized to other aspects of life in which violence is not yet endorsed or normalized. Military socialization regarding legitimate use of violence in some contexts may spillover to endorsement and perpetration of IPV as a conflict resolution technique within intimate relationships (Jones, 2012). Bradley (2007) tested whether cultural spillover may operate even after a military spouse has left active military service; however, the analysis showed no support for cultural spillover theory, in that veterans were not more likely to perpetrate IPV than non-veterans. Lysova and Straus (2021) tested cultural spillover theory utilizing data from 32 countries on dating violence, hypothesizing that agreement with socially approved forms of violence would be associated with perpetration of physical IPV. They found that both at the individual and national-level endorsement of “legitimate violence” was associated with IPV perpetration, supporting cultural spillover theory (Lysova & Straus, 2021).

#### Family Theory

Family theories consider the family, a system or unit where all members interact with each other, as a site where violence perpetration can be generated, whether through stressors that all family members experience or patterns of inter-relationships in the family (Chornesky, 2000; Murray, 2006; Zosky, 2006). Family system theory posits that a family is a dynamic social system with nested subsystems that can simultaneously impact one another (Pu & Rodriguez, 2021; Saint-Eloi Cadely et al., 2021). Cadely et al. (2021) found that having the same partner for a long time may lead to the emergence and escalation of extensive psychological IPV, and Baker et al. (2018) found that lower self-control in partners may lead to increased emotional and verbal aggression. In both cases, the relationship closeness within the couple affects the IPV outcome. In studies focused on IPV and parenting behavior, researchers found that since families are interdependent, parental conflict may spillover to the parent–child relationship. For instance, Pu and Rodriguez (2021) showed that in their sample spillover of conflict occurred bidirectionally between reported IPV experience and parent–child aggression risk for the mothers and unidirectionally from reported IPV experience to subsequent parent–child aggression risk for fathers. Finally, Rosen et al. (2001) and Schubert et al. (2002) examined the intergenerational transmission of violence from family of origin to IPV among couples. They applied Bowen’s (1966) theory of differentiation, according to which individuals who cannot separate their thoughts from emotions and have a lower tolerance for fluctuations in relationship closeness tend to lose themselves in a relationship. This concept of couple differentiation was found to be lower for partners with previous experiences of abuse and was consequently associated with higher IPV.

#### Peer Relations

Peer theory, accounting for peer relationships as potential predictors of women’s experiences of IPV, was addressed in seven included studies. Peer relation theories indicate that male peer relationships may be pervaded by gender and social norms supportive of IPV perpetration, and that perceptions of peers’ endorsement of abusive behaviors may lead to initiation or continuation of IPV perpetration. Schwartz et al. (2001) drew on peer support theory to bring the analysis of the motivation of the “motivated offender” from routine activity theory beyond sociodemographic variables and toward explanations that consider rape culture and male peer groups. They found that male peer relationships supportive of physical assault against women were associated with male sexual IPV perpetration on college campuses in Canada (Schwartz et al., 2001).

Beckmann et al. (2021), drawing on the theory of normative conduct, proposed that peer relationships and behaviors are primary predictors of dating violence perpetration. In their study of high-school students, they found that classroom-levels of dating violence were significantly associated with individual-level perpetration rates. They concluded that individual-level propensity toward IPV perpetration is impacted by aggregate peer behaviors, as well as association with physically aggressive peers (Beckmann et al., 2021). Mulawa et al. examined the influence of peer network on IPV perpetration among young men in Dar es Salaam, Tanzania, both quantitatively and qualitatively. In their qualitative research, they identified three key mechanisms through which men indicate that peer groups influence their perpetration of IPV: internalizing the norms of peer networks, feeling pressure to conform to these norms, and peers directly shaping the power dynamics of couples (Mulawa, Kajula et al., 2018). In their quantitative study, Mulawa et al. explored associations between peer gender norms, peer social cohesion, and IPV perpetration among men in Dar es Salaam, Tanzania. They found that peer network gender norms were significantly associated with IPV perpetration, and peer social cohesion moderated this relationship, with peer networks characterized by medium or high level of social cohesion displaying a significant association between inequitable peer gender norms and men’s risk of perpetrating IPV (Mulawa, Reyes et al., 2018).

#### Intersections Between Theories and the Ecological Framework

The ecological framework offers a means of comparing, contrasting, and integrating the sociological theories employed to explain IPV against women. The ecological framework conceptualizes predictors of IPV as operating at various levels of analysis: personal history, for example, witnessing violence as a child, micro-system, for example, the relationships and immediate context of the violence; predictors at this level include use of alcohol or male dominance within the family, exosystem, for example, the formal and informal social institutions and structures within which the micro-system is embedded; predictors at this level include low socioeconomic status and unemployment and macro-system, for example, the broader views and norms of society and culture; predictors at this level include rigid gender norms and social norms accepting use of violence against women (Heise, 1998).

As indicated in Table 4, sociological theories incorporate some account of structural influences on individual behaviors, as well as engaging with the exo- and macro-systems. Mapping specific sociological sub-theories onto the ecological framework can indicate potential causal pathways and mechanisms for further research. For example, poor self-control is an individual-level predictor of male perpetration of IPV. At the micro-system, levels of self-control may be impacted by alcohol-use, which, in turn, at the exosystem level, may be reinforced by social disorganization. At the macro-level, societal-wide beliefs about the legitimacy of violence and patterns of use of violence outside of intimate partner relationships—cultural theories of IPV—may shape patterns of social disorganization. Applying several sub-theories to different levels of the ecological framework indicates opportunities for theoretical synthesis to advance complex, multilevel, and reciprocal pathways between various predictors of IPV against women.

## Discussion

In this review and narrative synthesis of studies applying sociological theories to understanding predictors of IPV, 108 studies utilized a wide range of sociological theories to explain IPV against women. A common thread between all these theoretical approaches is the focus on structures, environment, and societal processes, often instead of or alongside accounts of individual-level risk or protective factors for female IPV experience or male IPV perpetration. In a notable number of studies, sociological theories are proposed alongside other theoretical approaches (psychological theories, feminist theories, and economic theories), often providing accounts of IPV explicitly or implicitly mirroring the ecological framework. As Table 4 indicates, most sub-theories engage with the individual-level as well as micro-system, and sometimes also exo- and macro-systems. Many researchers and theorists in the included studies argued that in the past, sociological approaches to understanding IPV had been sidelined in favor of other theoretical approaches, such as feminist and psychological theories, given perceptions of IPV as inherently different than other crimes. As Outlaw (2015) noted, many sociological theoretical approaches to criminal victimization have purposefully not addressed IPV, as it was perceived as a “conceptually different phenomenon” than other crimes, with key differences in terms of motivation and location of occurrence (Mannon, 1997; Outlaw, 2015). However, analysis of publication dates for the included studies indicates a significant increase in publications that employ sociological theories to account for predictors of IPV, demonstrating a growing interest in application of sociological theories to investigate predictors of IPV. Table 5 summarizes key findings and Table 6 presents implications for research and practice.

**Table 5.** Findings.

Key Findings
Sociological theories of predictors of IPV focus on social structures and their influence on dynamics and patterns of intimate partner violence (IPV)
This systematic review identified several sociological sub-theories, which account for IPV: criminological theories, neighborhood theory, cultural theory, family theory, and peer theory, all of which have overlapping concepts and concerns
Sociological theories address different levels of the ecological framework and have relevance to prevalence of IPV against women globally

**Table 6.** Implications.

Implications
*Research*: Sociological theories can be employed to frame and understand studies exploring predictors of intimate partner violence (IPV), as well as interventions designed to address social structures as a mean to prevent and reduce IPV Studies can draw upon several overlapping conceptual frameworks and underlying principles to understand male perpetration and women’s experience of IPV Sociological theories hold explanatory power for comprehending how, where, and why IPV occurs
*Practice*: Interventions focusing on relationships, family, and community implicitly or explicitly draw upon sociological theories of IPV and can be strengthened by developing theories of change or intervention mechanisms based on these overlapping theories *Practice*: Interventions to prevent and respond to IPV may draw upon sociological theories to develop, refine and implement interventions

In our narrative analysis, we provide description of studies categorized and labeled into sub-theories; however, a primary finding of the review is the considerable overlap between the sub-theories (Table 4), something some theorists in these studies recognized. Smith (1991) argued that peer relation theories emerge from sub-culture of violence theories, whereby a specific peer group of males who endorse and/ or perpetrate IPV act to encourage and support male IPV perpetration. How cultural spillover theory was operationalized sometimes overlapped with neighborhood theories—for example, Lysova and Straus’ (2021) test of cultural spillover theory, which hypothesized that endorsement of “legitimate violence” at the national level would also be associated with IPV perpetration, is similar to social contagion theory. Wright and Benson’s (2010) work on the immigrant paradox, which they identify as part of a sub-culture of violence theory, is very similar to neighborhood theories that examine the association between concentration of immigrant populations and IPV perpetration.

Each broad theory and sub-theory outlined in this article is partial and limited to fully explain the complex phenomenon of IPV, and research and theoretical development could fruitfully draw on these interconnections to strengthen specific sub-theories. For example, routine activity theory employs a central assumption—that “capable guardianship” exists, and would deter or prevent male IPV perpetration (Mannon, 1997). Linkages to evidence on the role of peer behaviors, primarily described only in the few articles focused on peer theory, may strengthen the concept of capable guardianship. Guardianship may not be effective or available not only due to individuals not being present at the time of IPV perpetration, but also due to potential guardians, for example, peers, community members, actually endorsing IPV perpetration. Cultural spillover theory also sheds light on how and why effective guardianship may exist in some contexts and not in others; in that, wider cultural beliefs and practices around violence can spillover into intimate relationships, such that effective bystander action may be more difficult. The spatial analyses in some of the studies utilizing neighborhood theories could be employed to understand how neighborhood structure, or structure of other institutions, such as college campuses, enables or deters capable guardianship. Moreover, the key insight of routine activity theory regarding the importance of capable guardianship to prevent IPV perpetration could be strengthened by drawing on newer evidence regarding the role of social and gender norms in impacting levels of IPV (Heise & Kotsadam, 2015). Recent efforts testing effective IPV perpetration interventions have found that curricula focused on bystander education models and various behavior change techniques can effectively reduce male IPV perpetration and increase prosocial bystander behaviors (Yount et al., 2023).

Evidence from studies employing sociological theories is extremely skewed toward high-income contexts. We found that only 21% of studies that utilized quantitative empirical data were conducted in one or multiple LMICs, and nearly 40% of included studies only included data from the United States. There is overall lack of consideration of differences in cultural or ethnic backgrounds of male perpetrators of IPV, and how these may impact patterns of perpetration, which is a limitation of the existing evidence-base. The paucity of studies employing sociological theories to understand predictors of IPV against women in LMICs is striking, given evidence that variations in prevalence of IPV across LMICs is associated with macro-level structural factors (Heise & Kotsadam, 2015). In particular, only one study in Santiago, Chile utilized neighborhood theory to interpret variations in IPV experience among women (Kim et al., 2013). Neighborhood theories, particularly the constructs of social disorganizations and social cohesion, have rarely been employed to understand IPV against women in LMICs. Even in studies employing neighborhood theories in high-income contexts, there is a bias toward urban settings. When Morgan and Jasinski (2017) extended social disorganization theory to examine neighborhood and county effects in urban and rural areas in Illinois, they found strong support for social disorganization theory in urban neighborhoods but minimal support for social disorganization theory in rural counties. Yet, this is only one of the few studies applying neighborhood theories to rural areas, and further investigation should seek whether some concepts or variables may be applicable there too.

This systematic review indicates that there are several compelling sociological theories that can be employed to guide study design and data analysis in the field of research on predictors of male-perpetrated IPV. While this review did not focus on IPV prevention interventions, it is evident that program designers and implementers could also draw on this body of theories to inform developing theories of change or intervention content. Moreover, this review identified intersections between different sociological sub-theories, and between sociological theories and other theoretical explanations of male-perpetrated IPV, such as feminist theories and economic theories. Understanding of these intersections can be used to inform research and intervention design based on combined, integrated theories, which can address the complex and multi-faceted predictors of IPV.

### Limitations

During the search, a manuscript not mentioning “theory” in the abstract would not be included in the review. This may have excluded studies that employed relevant sociological theories. We restricted the included studies to peer-reviewed journal articles published in English. Given the search strategy, we cannot determine that the included articles represent all empirical data on these sociological theories. However, we provide a comprehensive overview of the key tenets of each sub-theory and a critical analysis of the logic and utility of these theories in identifying and explaining predictors of IPV against women. The review focuses only on sociological theories accounting for male-perpetrated IPV, which may not be generalizable to female-perpetrated IPV or IPV within same-sex relationships.

## Conclusion

There is a long and rich history of sociological theories addressing dynamics within relationships and families. IPV has been viewed as a different social phenomenon—largely due to being perceived as “private”—within some strains of sociological theory. However, over the past three decades, sociological interest in explaining and understanding IPV has increased significantly. This systematic review fills a gap in theoretical syntheses of sociological theories of predictors of male-perpetrated IPV against women. Also, it provides critical analysis of how these theories overlap and intersect. While sociological theories may not be able to fully explain all aspects of dynamics of male-perpetrated IPV against women, this overview indicates that there are several compelling components of sociological theory that hold explanatory power for comprehending how, where, and why IPV occurs.
